# Supplementary material for: Topics Mentioned by Stroke Patients during Supportive Phone Calls—Implications for Individualized Aftercare Programs
Source: Healthcare (Basel). 2022 Nov 29;10(12):2394. doi: 10.3390/healthcare10122394 (PMC9777582; doi:10.3390/healthcare10122394)
Supplement: Supplementary file 1 [file healthcare-10-02394-s001.zip › healthcare-2021911-supplementary.pdf]

**Supplements**

Table S1. Conversation topics mentioned by all patients during phone call. Topics are ordered by descending number of occurrence.

| Count | Topic | % |
|---|---|---|
| 106 | rehabilitation | 67.5 |
| 49 | specialist physician | 31.2 |
| 42 | symptoms | 26.8 |
| 33 | medication | 21.0 |
| 31 | outpatient care | 19.7 |
| 31 | therapy | 19.7 |
| 29 | aids and appliances | 18.5 |
| 29 | blood pressure | 18.5 |
| 26 | driving license | 16.6 |
| 26 | occupation | 16.6 |
| 21 | disability | 13.4 |
| 19 | home environment | 12.1 |
| 17 | general practicioner | 10.8 |
| 15 | risk factors | 9.6 |
| 12 | sports | 7.6 |
| 7 | other medical condition | 4.5 |
| 6 | physiotherapy | 3.8 |
| 6 | speech therapy | 3.8 |
| 5 | nutrition | 3.2 |
| 4 | depression | 2.5 |
| 4 | finances | 2.5 |
| 4 | health insurance | 2.5 |
| 4 | smoking | 2.5 |
| 3 | corona virus | 1.9 |
| 3 | exercise | 1.9 |
| 3 | patent foramen oval | 1.9 |
| 2 | alcohol | 1.3 |
| 2 | body weight | 1.3 |

| | | |
|---|---|---|
| 2 | daily routine | 1.3 |
| 2 | diabetes | 1.3 |
| 2 | electrocardiogramm | 1.3 |
| 2 | leisure time | 1.3 |
| 2 | life style | 1.3 |
| 2 | medical care | 1.3 |
| 2 | relatives / care takers | 1.3 |
| 2 | sleep | 1.3 |
| 1 | anxiety | 0.6 |
| 1 | atrial fibrillation | 0.6 |
| 1 | concentration | 0.6 |
| 1 | disease awareness | 0.6 |
| 1 | fatigue | 0.6 |
| 1 | hospitalisation | 0.6 |
| 1 | memory | 0.6 |
| 1 | mobility | 0.6 |
| 1 | occupational therapy | 0.6 |
| 1 | personal performance | 0.6 |
| 1 | psychotherapy | 0.6 |
| 1 | self-help group | 0.6 |
| 1 | sleep apnea | 0.6 |

Table S2. Adjusted odds ratios for mentioning specific topics.

| | female sex | age | NIHSS admission | no. secondary diagnoses | no. risk factors | days in hospital | NIHSS discharge | mRS discharge | no. meds discharge | no rehabilitation | inpatient rehabilitation | multi-person household |
|---|---|---|---|---|---|---|---|---|---|---|---|---|
| **rehabilitation** | 1.34 (0.59-3.12) | 0.99 (0.95-1.02) | 1.05 (0.93-1.20) | 1.22 (1.00-1.51) | 0.93 (0.76-1.24) | 0.98 (0.91-1.06) | 1.12 (0.86-1.48) | 0.57 (0.33-0.96) | 0.94 (0.80-1.10) | 0.78 (0.23-2.72) | 1.68 (0.63-4.55) | 0.97 (0.36-2.49) |
| **specialist physician** | 0.55 (0.23-1.29) | 1.00 (0.97-1.04) | 1.07 (0.96-1.20) | 1.11 (0.94-1.33) | 1.24 (0.95-1.65) | 0.97 (0.88-1.05) | 0.73 (0.52-0.98) | 1.16 (0.69-1.97) | 0.99 (0.84-1.15) | 1.85 (0.48-6.90) | 1.11 (0.40-3.12) | 2.64 (0.93-8.53) |
| **symptoms** | 1.50 (0.61-3.69) | 0.96 (0.93-1.00) | 1.08 (0.97-1.20) | 0.97 (0.78-1.17) | 1.45 (1.10-1.98) | 0.99 (0.90-1.07) | 1.09 (0.82-1.43) | 0.41 (0.22-0.72) | 1.09 (0.92-1.30) | 0.66 (0.09-3.19) | 1.24 (0.45-3.54) | 1.05 (0.37-3.14) |
| **medication** | 0.34 (0.11-0.91) | 1.02 (0.98-1.06) | 0.98 (0.84-1.12) | 1.09 (0.90-1.32) | 1.21 (0.90-1.65) | 1.00 (0.90-1.08) | 0.66 (0.43-0.97) | 1.31 (0.73-2.39) | 0.95 (0.78-1.12) | 2.06 (0.51-8.20) | 0.75 (0.23-2.46) | 1.01 (0.33-3.30) |
| **outpatient care** | 5.98 (1.80-23.52) | 1.09 (1.03-1.17) | 1.00 (0.84-1.17) | 0.99 (0.81-1.21) | 1.15 (0.78-1.69) | 1.14 (1.01-1.28) | 1.31 (0.88-2.06) | 1.68 (0.72-4.20) | 1.22 (0.99-1.53) | 1.24 (0.11-1.27) | 1.40 (0.23-1.04) | 0.93 (0.25-3.60) |
| **therapy** | 0.60 (0.22-1.50) | 1.02 (0.98-1.06) | 1.08 (0.96-1.21) | 0.98 (0.80-1.17) | 1.06 (0.79-1.42) | 0.97 (0.87-1.06) | 1.16 (0.89-1.53) | 0.70 (0.39-1.23) | 0.89 (0.73-1.06) | 2.17 (0.46-9.41) | 1.72 (0.57-5.48) | 1.02 (0.36-3.07) |
| **aids and appliances** | 2.30 (0.80-6.81) | 1.07 (1.02-1.14) | 1.05 (0.89-1.22) | 0.84 (0.64-1.04) | 1.08 (0.73-1.57) | 1.01 (0.88-1.13) | 0.69 (0.47-0.96) | 5.35 (2.34-14.65) | 1.21 (1.01-1.47) | 0.71 (0.10-4.20) | 0.57 (0.12-2.68) | 1.56 (0.45-5.95) |
| **blood pressure** | 1.36 (0.51-3.60) | 1.00 (0.97-1.05) | 0.85 (0.69-0.99) | 0.86 (0.68-1.07) | 1.50 (1.10-2.13) | 1.01 (0.92-1.10) | 1.07 (0.74-1.51) | 1.03 (0.57-1.89) | 0.98 (0.81-1.16) | 1.32 (0.24-6.09) | 2.25 (0.75-7.25) | 4.71 (1.30-23.30) |
| **driving license** | 0.40 (0.11-1.28) | 1.00 (0.96-1.04) | 1.02 (0.88-1.17) | 0.71 (0.53-0.93) | 1.40 (0.97-2.04) | 1.06 (0.97-1.16) | 0.53 (0.31-0.81) | 2.92 (1.48-6.21) | 0.97 (0.77-1.18) | 2.61 (0.55-12.35) | 1.48 (0.41-5.58) | 2.48 (0.64-12.72) |
| **occupation** | 0.62 (0.16-2.07) | 0.89 (0.83-0.94) | 1.04 (0.89-1.19) | 0.98 (0.66-1.38) | 1.22 (0.77-1.89) | 0.95 (0.83-1.05) | 0.93 (0.61-1.37) | 1.03 (0.49-2.21) | 0.75 (0.54-1.00) | 0.91 (0.10-6.15) | 1.10 (0.30-4.12) | 0.93 (0.21-4.58) |
| **disability** | 1.23 (0.35-4.20) | 1.02 (0.97-1.08) | 1.01 (0.85-1.17) | 0.80 (0.57-1.04) | 0.93 (0.56-1.46) | 0.98 (0.84-1.09) | 1.08 (0.78-1.56) | 3.04 (1.32-1.16) | 0.99 (0.78-1.23) | 0.00 | 1.13 (0.26-5.09) | 3.66 (0.80-2.31) |
| **home environment** | 1.91 (0.58-6.47) | 1.09 (1.02-1.17) | 1.18 (1.00-1.39o) | 1.03 (0.82-1.27) | 0.75 (0.46-1.12) | 0.93 (0.78-1.07) | 0.94 (0.67-1.29) | 1.95 (0.86-4.76) | 1.05 (0.81-1.32) | 0.19 (0.01-1.76) | 0.25 (0.04-1.37) | 0.39 (0.10-1.48) |
| **general practicioner** | 0.71 (0.17-2.59) | 0.99 (0.94-1.04) | 1.02 (0.85-1.17) | 0.95 (0.67-1.26) | 0.73 (0.40-1.20) | 0.96 (0.82-1.07) | 1.26 (0.86-1.82) | 0.57 (0.24-1.23) | 0.99 (0.74-1.25) | 0.00 | 1.51 (0.37-6.70) | 1.85 (0.40-13.39) |
| **risk factors** | 1.69 (0.51-5.64) | 0.96 (0.91-1.01) | 0.85 (0.61-1.06) | 1.06 (0.77-1.40) | 1.11 (0.72-1.67) | 0.93 (0.78-1.06) | 0.81 (0.45-1.33) | 1.93 (0.87-4.67) | 1.01 (0.78-1.29) | 0.70 (0.08-4.67) | 0.70 (0.14-3.29) | 0.82 (0.20-3.75) |

Statistical relationship between clinical and socio-demographic variables during the hospital stay and mentioned topics two months after the event. Table shows adjusted odds ratios (aOR) with 95%-confidence interval calculated by a multivariate logistic regression model with conversation topic as a dependent variable (rows) and clinical and socio-demographic factors as explaining variables (columns). Cells with significant aORs are highlighted.
